# Association between a urinary biomarker for exposure to PAH and blood level of the acute phase protein serum amyloid A in coke oven workers

**DOI:** 10.1186/s12940-019-0523-1

**Published:** 2019-09-02

**Authors:** Niels Hadrup, Danuta Mielżyńska-Švach, Agnieszka Kozłowska, Manuela Campisi, Sofia Pavanello, Ulla Vogel

**Affiliations:** 1 National Research Centre for the Working Environment, DK-2100 Copenhagen, Denmark; 2 Institute of Occupational Medicine and Environmental Health, Sosnowiec, Poland; 3 Witold Pilecki State School of Higher Education, Nursing Institute, Oświęcim, Poland; 4 Department of Cardiac, Thoracic and Vascular Sciences, University of Padova, Padova, Italy

**Keywords:** Acute phase response, Particles, Pulmonary, Toxicity, Serum amyloid A

## Abstract

**Background:**

Coke oven workers are exposed to both free and particle bound PAH. Through this exposure, the workers may be at increased risk of cardiovascular diseases. Systemic levels of acute phase response proteins have been linked to cardiovascular disease in epidemiological studies, suggesting it as a marker of these conditions. The aim of this study was to assess whether there was association between PAH exposure and the blood level of the acute phase inflammatory response marker serum amyloid A (SAA) in coke oven workers.

**Methods:**

A total of 87 male Polish coke oven workers from two different plants comprised the study population. Exposure was assessed by means of the individual post-shift urinary excretion of 1-hydroxypyrene, as internal dose of short-term PAH exposure, and by anti-benzo[a]pyrene diolepoxide (anti-B[a]PDE)-DNA), as a biomarker of long-term PAH exposure. Blood levels of acute phase proteins SAA and CRP were measured by immunoassay. C-reactive protein (CRP) levels were included to adjust for baseline levels of SAA.

**Results:**

Multiple linear regression showed that the major determinants of increased SAA levels were urinary 1-hydroxypyrene (beta = 0.56, *p* = 0.030) and serum CRP levels (beta = 7.08; *p* < 0.0001) whereas anti-B[a]PDE-DNA, the GSTM1 detoxifying genotype, diet, and smoking were not associated with SAA levels.

**Conclusions:**

Urinary 1-hydroxypyrene as biomarker of short-term PAH exposure and serum levels of CRP were predictive of serum levels of SAA in coke oven workers. Our data suggest that exposure of coke oven workers to PAH can lead to increased systemic acute response and therefore potentially increased risk of cardiovascular disease.

**Electronic supplementary material:**

The online version of this article (10.1186/s12940-019-0523-1) contains supplementary material, which is available to authorized users.

## Background

Coke oven workers are exposed to polluted air. This polluted air consists of a particle and a gaseous phase both containing a long range of different substances including polycyclic aromatic hydrocarbons (PAH). The exposure of coke oven workers to polluted air likely poses a risk to their health. Air pollution has previously been associated to such diseases as cancer and cardiovascular diseases [1, 2]. Exposure to PAH in the American population has been reported to be associated to cardiovascular disease [3, 4] and to peripheral arterial disease [5] in addition, PAH was found to contribute to the increased carotid intima-media thickness - an atherosclerosis marker - in taxi drivers [6]. Pulmonary exposure to particulate air pollution has been associated with increased risk of cardiovascular morbidity and mortality [7–9].

The acute phase response is activated during inflammation. The blood levels of acute phase proteins Serum amyloid-A (SAA) and C-reactive protein (CRP) are suggested to be predictive of cardiovascular disease risk in humans [10]. SAA is causally implicated in atherosclerosis by promoting formation of foam cells and by increased formation of plaques in aorta [11–19], whereas the function of CRP is less clear [20]. PAH exposure has been reported to be associated with increased CRP levels in humans [21]; and it has been hypothesised that particle exposure may cause cardiovascular disease through particle-mediated acute phase response and lung inflammation [22, 23]. In mice, airway exposure to insoluble particles induces a dose-dependent pulmonary acute phase response, a complex systemic response characterised by alterations in blood levels of Serum Amyloid A (SAA) [23–32]. Recently, inhalation of ZnO nanoparticles was shown to induce dose-dependent acute phase response in terms of SAA and CRP blood levels in human volunteers [33]. ZnO dissolves when phagocytized by macrophages. This has provided important evidence for particle-induced acute phase response in humans [34], but evidence for induction of acute phase response by insoluble particles is still needed.

In this work we hypothesised that the acute phase response was activated after exposure to coke oven emissions. To investigate this, we needed an appropriate biomarker of exposure. We assessed PAH as a marker of airway pollution because: 1) PAH is an important constituent of coke oven emissions [35–37]; and 2) it is in itself known to be toxic – as described above. In the current work we assessed short (hours to days) and long-term (months) PAH exposure by measuring urinary excretion of the pyrene metabolite 1-hydroxypyrene, a commonly used marker of the internal dose of PAH [37–42] and DNA adduct levels of benzo[a]pyrene diolepoxide (anti-B[a]PDE)-DNA) adduct in blood cells, a marker of the effective long-term PAH exposure.

Thus to investigate the association between the exposure to coke oven air pollution and the acute phase response, we measured the blood concentration of SAA and conducted multiple linear regression using the PAH biomarkers 1-hydroxypyrene and anti-B[a]PDE)-DNA as well as the GSTM1 genotype, smoking, charcoaled meat consumption, and C-reactive protein (CRP) levels as explanatory variables. The GSTM1 null genotype was included as it has previously been shown to be a risk factor for anti-B[a]PDE-DNA adduct formation [43, 44]. Smoking and charcoaled meat consumption were included in the analysis to adjust for PAH exposure through these sources. C-reactive protein (CRP) levels were included to adjust for baseline levels of SAA [45].

## Methods

### Workers included in the investigation

Eighty-seven male Polish coke oven workers were enrolled from two plants as described previously [43, 46]. For each subject, data regarding age, tobacco smoking and charcoal-broiled meat consumption, occupational and environmental exposure to PAH, personal behaviour and the possible use of coal tar-based ointment, shampoo or soap, were collected by means of a questionnaire administered by trained interviewers. The characteristics of the study group are summarised in Table 1. All participants gave their informed consent. All information regarding participants was rendered anonymous after collection of data, urine and blood samples. The study design was reviewed and approved by the appropriate Ethics Committee of the Institute of Occupational Medicine and Environmental Health in Sosnowiec (Poland).

**Table 1:** General characteristics of enrolled coke oven workers

Variable [unit]	
Number of subject [N (%)]	
Female	0 (0)
Male	87 (100)
Age in years [mean ± SD, range]	39 ± 10 (22–55)
GSTM1 genotypes [N (%)]	
Active	58 (66)
*0/*0	29 (33)
Current smoking habits^a^ [N (%)]	
Non-smokers	34 (39)
Smokers	53 (61)
Level of smoking in smokers (cigarettes/day) [mean ± SD, range]	23 ± 7 (8–40)
Diet^b^[N (%)]	
No≥once a week	22 (25)
No<once a week	65 (75)

^a^Smokers are defined as current smokers

^b^Numbers of subjects with charcoaled meat consumption ≥ or < once a week

### Sample collection

Urine (50 mL) and blood (20 mL) samples were collected from all subjects between March and June 2002. Samples from workers were collected at the end of the work-shift, after at least 3 consecutive days of work. Within 4 h of blood collection, mononuclear lymphocyte plus monocyte fractions (LMF) were isolated in Ficoll separating solution (Seromed, Berlin, Germany), as previously described [47]. LMF, serum and urine samples were kept frozen at − 80 °C until their transport to the Department of Environmental Medicine and Public Health in Padova (Italy) for further analysis.

### Determination of 1-hydroxypyrene, anti-B[a]PDE-DNA adducts, and GSTM1 genotypes

PAH exposure (occupational, environmental and dietary sources) was evaluated by means of the post-shift urinary excretion of 1-hydroxypyrene, normalised to creatinine (μmol1-hydroxypyrene /mol creatinine) and anti-B[a]PDE-DNA adduct levels in the blood fraction consisting of both mononuclear lymphocytes and monocytes. 1-hydroxypyrene and anti-B[a]PDE-DNA adducts were previously reported [43]. GSTM1 genotyping data were previously published [43]. Briefly, 1-hydroxypyrene was determined in urine samples as formerly described [47], following the original method of Jongeneelen [48]. The levels were expressed per moles of urinary creatinine, determined according to the Boehringer–Mannheim colorimetric test, based on the reaction of creatinine with picrate in alkaline medium. As proposed by Jongeneelen [48], we considered the urinary 1-hydroxypyrene value of 2.28 μmol/mol creatinine as the biological exposure index (BEI). It was calculated that a concentration of 2.28 μmol 1-hydroxypyrene/mol creatinine after a 3-day working period in the urine of coke oven workers equals the airborne threshold limit value (TLV) of coal tar pitch volatiles (CTPV), i.e. 0.2 mg/m^3^ of ‘benzene-soluble matter’ [49]. After DNA extraction, anti-B[a]PDE-DNA adducts were detected by HPLC/fluorescence analysis of anti-BaP tetrols (tetrol I-1, see abbreviations) released upon acid hydrolysis of DNA samples. HPLC/fluorescence analysis of anti-B[a]PDE-DNA adducts was carried out as previously described [50], and is identical to that reported by [51] with some modifications mainly regarding the entire automation of the HPLC analysis. Briefly, a total amount of at least 100 μg of DNA from LMF was used for each analysis. Many precautions were taken to avoid the presence of fluorescent contaminates: the absence of any fluorescent material in the purified HCl was checked by HPLC; tubes, HPLC syringes and other equipment were washed many times with HPLC purity-grade methanol, and a blank injection was performed before each sample was subjected to HPLC analysis. The fluorescence excitation wavelength was set at 344 nm and the emission wavelength at 398 nm. The level of B[a]P-tetrol-I-1 was determined by comparison with a standard curve generated from the fluorescence areas of an authentic B[a]P-tetrol-I-1 standard (purchased from NCI Chemical Carcinogen Reference Standard Repository, Kansas City, MO, USA), analysed prior to and immediately following the analysis of each set of samples. Calibration curves were made with DNA from calf thymus, alone (background) and spiked with 2, 4, 10, 20, 40 and 80 pg of B[a]P-tetrol-I-1. These standard solutions were then treated in the same way as the tested samples (hydrolysed in 0.1 N HCl at 90 °C for 4 h). The minimum correlation coefficient was 0.9998 and the mean coefficient of variation for analyses repeated on different days was 10%. The detection threshold of B[a]P-tetrol-I-1 was 1 pg (signal/noise > 3) so that, in the present study, with 100 μg DNA, this assay can measure 1 adduct/10^8^ nucleotides (1 fmol/μg DNA = 30 adducts/10^8^ nucleotides). A multiplex polymerase chain reaction (PCR) method was used to detect the presence or absence of the GSTM1 gene, according to the protocol formerly described [52]. This PCR method applied, in the same amplification mixture, GSTM1-specific primer pairs and a primer pair for β-globin, as an internal positive PCR control. β-Globin (285 bp) and GSTM1 (215 bp) amplification products were resolved in an ethidium bromide-stained 2% agarose gel. The absence of the GSTM1-specific fragment indicated the corresponding null genotype (*0/*0), the presence of the fragment correspond with the *1/*1 and *0/*l genotypes, whereas the β-globin-specific fragment confirmed the presence of amplifiable DNA in the reaction mixture.

### Determination of acute phase proteins

Upon blood sampling SAA and CRP levels were determined by Enzyme-linked Immunosorbent assay (ELISA) as previously described [53]. In brief, SAA was determined in serum samples with kit product number KHA0011 (Invitrogen CA, USA), as specified by the manufacturer. The reported detection limit (LOD) was 4 ng/mL. Recombinant human SAA1 supplied with the kit was included as positive control in all measurement runs. CRP was determined with a kit from IBL International GMBH (prod number EU59151, Hamburg, Germany). The LOD was 20 ng/mL. All SAA and CRP measurements were above LOD. Human CRP was used as positive control in all measurement runs (code 85/506, NIBSC).

### Statistics

Linear multiple regression analyses were done using the BMDP software package [54]. The analysis comprised, SAA as a dependent variable with the following explanatory variables: 1. Urinary excretion of 1-hydroxypyrene, 2. Anti-B[a]PDE-DNA-adducts, 3. GSTM1 status, 4. CRP. 5. Smoking habits, 6. Diet habits. Smoking was included as 1 or 0, based on whether the worker was current smoker or a non-smoker. Diet was included as 1 or 0 based on whether charcoaled meat consumption was more or less than once per week, respectively. *P-*values were calculated for each explanatory variable. The slope (β) as well as the test statistics (T = β/standard deviation of β) were reported. The F-test was calculated to determine the overall significance of the test. In addition the above tests were done in the absence of CRP as an explanatory variable. And CRP was tested as dependent variable with explanatory variables 1-hydroxypyrene, 2. Anti-B[a]PDE-DNA-adducts, 3. GSTM1 status, 4. SAA. 5. Smoking habits, 6. Diet habits. And this latter test was also done excluding SAA as an explanatory variable.

The serum levels of SAA and CRP were also tested for changes between 3 ranges of the urine level of 1-hydroxypyrene (I ≤ 2.30 /μmoles/mole creatinine; II > 2.30 ≤ 7.04, and III > 7.04). This was done by the Mann-Whitney *U* test.

## Results

As reported previously [43], coke-oven workers were heavily exposed to PAH, as the urinary 1-hydroxypyrene concentrations in 70, 90 and 85% of the workers exceeded 2.28 μmol/mol creatinine, 1.0 μmol/mol creatinine and 2.5 μg/L, respectively. The two first values are the biological exposure index (BEI) proposed by Jongeneelen in 1992 and 2014 [38, 39]. The third value is the BEI proposed by ACGIH in 2016 [55]. The 2.28 value corresponds to the post-shift excretion value after an environmental exposure to the airborne threshold limit value of coal tar pitch volatiles (i.e., 0.2 mg/m^3^ of “benzene soluble matter,” ACGIH). For this value a relative risk (RR) of 1.3 for lung cancer has been estimated for coke oven workers [38]. The BEI of 2.5 μg/L proposed by ACGIH in 2016 [55] is adjusted for the pyrene to benzo(a)pyrene ratio of the PAH mixture to which workers are exposed. This value corresponds to 1.3 μmol/mol creatinine at the usual urinary creatinine level of 1 g/L and is therefore very similar to a value of 1.4 μmol/mol creatinine proposed by Buchet et al.; based on the minimum level of urinary 1-hydroxypyrene associated with increased frequency of sister chromatid exchanges in peripheral blood lymphocytes of non-smokers [56]. Moreover, anti-B[a]PDE-DNA adducts were detected in the blood of almost all of the coke oven workers (98%, *n* = 85).

SAA and CRP levels correlated closely (*R*^*2*^ = 0.74) but varied greatly between individuals (Table 2). As baseline levels vary significantly between individuals due to biosynthesis in adipose tissue [45, 57, 58], CRP levels were used as a biomarker for baseline levels of acute phase response as previously described [45]. Linear multiple regression (Table 3) showed that the major determinants of SAA levels were urinary 1-hydroxypyrene (beta = 0.56, *p* = 0.03) and CRP serum levels (beta = 7.08; *p* < 0.0001) whereas anti-B[a]PDE-DNA adducts, the GSTM1 genotype, diet and smoking did not predict SAA levels. When CRP was excluded from the analysis there was no effect of 1-hydroxypyrene, but an effect of GSTM1 (*p* = 0.03) (Additional file 1: Table S1). The determinants of CRP levels were urinary 1-hydroxypyrene urinary 1-hydroxypyrene (beta = − 0.08, *p* = 0.01) and CRP serum levels (beta = 0.003; *p* < 0.0001) whereas anti-B[a]PDE-DNA adducts, the GSTM1 genotype, diet and smoking did not predict CRP levels (Table 4). When SAA was excluded from the analysis there was no effect of any of the explanatory variables (Additional file 2: Table S2). Concerning the potential relation of SAA, CRP, and 1-hydroxypyrene when not tested by multiple linear regression, the serum levels of SAA and CRP were not statistically related to the three categories of 1-hydroxypyrene in urine, when tested by the Mann-Whitney *U* test (Table 5).

**Table 2:** Levels of biomarkers of exposure and effect

Biomarkers/assay [unit]	Median	Mean ± SD	Range	N (%) above the limit value
1-hydroxypyrene urine concentration (μmol/mol creatinine)^a^	4.31	6.81 ± 7.40	0.25–31.4	60 (70)
Anti-B[a]PDE-DNA (adducts/ 10^8^ nucleotides)^b^	2.89	4.03 ± 3.65	1.00–27.4	85 (98)
SAA (μg/mL)	3.07	34.18 ± 180.14	0.36–1200	
CRP (μg/mL)	0.36	0.95 ± 2.73	0.4–24.3	

^a^Short- PAH exposure was evaluated by means of the urinary excretion of 1-hydroxypyrene. 1-hydroxypyrene levels were reported previously [43]

^b^Long-term PAH exposure was evaluated by means of anti-B[a]PDE-DNA adducts. Positive were workers with detectable adducts (> 1 adducts/10^8^ nucleotides). Anti-B[a]PDE-DNA adduct levels were reported previously [43]

**Table 3:** Predictors of SAA levels. Multiple linear regression analysis of influence of PAH exposure evaluated by urinary excretion of 1-hydroxypyrene, anti-B[a]PDE-DNA adduct levels in blood cells, GSTM1, CRP, smoking status and diet habits on serum SAA levels in coke oven workers (*n* = 87)

	1-hydroxypyrene	anti-B[a]PDE-DNA adducts	GSTM1^a^	CRP	Smoking^b^	Diet^c^
β^d^	0.56	−0.39	−1.05	7.08	−1.14	0.43
T^d^	2.20	0.86	1.61	14.5	1.88	0.70
*p*-value^d^	**0.030**	0.391	0.110	**< 0.0001**	0.063	0.487

^a^GSTM1 genotypes was treated as dichotomous variables: GSTM1 = 1 or 0, Active or *0/*0

^b^Smoking = 1 or 0, current smokers or non-smokers

^c^Diet = 1 or 0, charcoaled meat consumption more or less than once per week, respectively

^d^The F test gave an F of 38.6 and a *p* value of < 0.0001. β is the slope. T is the test statistics β/standard deviation of β

Bold font highlights statistically significant values

**Table 4:** Predictors of CRP levels. Multiple linear regression analysis of influence of PAH exposure evaluated by urinary excretion of 1-hydroxypyrene, anti-B[a]PDE-DNA adduct levels in blood cells, GSTM1, SAA, smoking status and diet habits on serum CRP levels in coke oven workers (*n* = 87)

	1-hydroxypyrene	anti-B[a]PDE-DNA adducts	GSTM1^a^	SAA	Smoking^b^	Diet^c^
β^d^	−0.08	0.01	0.02	0.003	0.14	−0.08
T^d^	−2.51	0.26	0.21	12.92	1.83	−0.94
*p*-value^d^	**0.014**	0.793	0.834	**< 0.0001**	0.072	0.348

^a^GSTM1 genotypes was treated as dichotomous variables: GSTM1 = 1 or 0, Active or *0/*0

^b^Smoking = 1 or 0, current smokers or non-smokers

^c^Diet = 1 or 0, charcoaled meat consumption more or less than once per week, respectively

^d^The F test gave an F of 30.04 and a *p* value of < 0.0001. β is the slope. T is the test statistics β/standard deviation of β

Bold font highlights statistically significant values

**Table 5:** Serum levels of SAA and CRP in relation to 1-hydroxypyrene urine levels

1-hydroxypyrene (μmoles/mole creatinine)	n	SAA Median(range)	CRP Median(range)
I ≤ 2.30	29	1.87 (0.62-34.6)	0,42 (0,04-24,3)
II > 2.30 ≤ 7.04	29	1,95 (0,60-6,66)	0,45 (0,06–2.00)
III > 7.04	29	1,67 (0,79-34,6)	0,33 (0,08-8,51)

The serum levels of SAA and CRP were not significantly different inbetween the different ranges of 1-hydroxypyrene - when tested by the Mann-Whitney *U* test

## Discussion

In the present study, we wanted to assess the potential association between coke oven occupational exposure to PAH and a marker of systemic acute phase response. Short-term exposure was assessed by urinary 1-hydroxypyrene excretion whereas long-term exposure was assessed by anti-B[a]PDE-DNA adduct levels in blood cells.

By multiple linear regression, urinary 1-hydroxypyrene was positively associated with SAA levels with a *p-*value of 0.03 and a slope of 0.6. At the same time, CRP was also associated with SAA with a *p*-value of less than 0.0001 and a slope of 7.1. This suggests that there is a link between PAH exposure and acute phase reaction induction in these workers, when adjusting for the association of CRP. CRP was inter-related to hydroxypyrene as the slope of 1-hydroxypyrene is different in the presence and absence of CRP in the analysis (Table 4 and Additional file 1: Table S1). Therefore it is reasonable to include CRP as an explanatory variable when analysing the association between PAH exposure and 1-hydroxypyrene to adjust for its variation. In addition, in a study on Finnish children and young adults randomly chosen from the national population registers of a number of Finnish cities, it was found that SAA correlated with CRP, waist circumference, and leptin and with different additional factors specifically in men and women [45]. Based on this, the inclusion of CRP as an explanatory variable adjusts for inter-individual variation in the baseline levels of SAA. As a side note, the serum levels of SAA and CRP were not statistically related to the three categories of 1-hydroxypyrene in urine, when tested by the Mann-Whitney *U* test (Table 5), reflecting that the association to 1-hydroxypyrene is only seen when analysing by multiple linear regression simultaneously taking more explanatory variables into account.

SAA and CRP levels correlated closely (*R*^*2*^ = 0.74) and CRP had, as an explanatory variable of the SAA serum level, a multiple linear regression slope of 7. SAA and CRP blood levels have previously been shown to be closely associated in humans, e.g. in [59–61]. This shows that these two acute phase proteins to some extend are simultaneously regulated in the human inflammatory response. However, they exert different functions. CRP acts as a nonspecific opsonin to enhance phagocytosis [62] and SAA has important functions in especially modulation of HDL-mediated cholesterol transport [63]. During an acute phase response, SAA replaces Apo-A1 in HDL, and by this reverses the cholesterol flow, thereby promoting foam cell formation of peripheral macrophages and increased formation of atherosclerotic plaques [11, 18, 19]. Thus, although co-regulated, these two proteins have distinct roles in the acute phase response justifying that they are analysed separately for their involvement in diseases and mechanisms.

Concerning the time course of induction of the acute phase response, our results suggest that 1-hydroxypyrene was predictive of SAA, whereas anti-B[a]PDE-DNA adducts, a biomarker of long-term exposure, was not predictive of SAA levels. This is in agreement with the time course for induction of the acute phase responses in animal studies following airway exposure to carbon nanoparticles and diesel exhaust particles [23, 24, 64, 65]. Likewise, the GSTM1 null genotype has previously been shown to be a risk factor for anti-B[a]PDE-DNA adduct formation [43, 44] and was also not predictive of the SAA levels.

It can be discussed whether the effect of PAH on SAA is mediated by the particular fraction and its exposure to the lungs. Concerning the pathway of lung exposure, this is likely the most pronounced exposure pathway in coke oven workers. However, PAH can be also taken up over intact skin and in the gastrointestinal tract, and thus dermal and dietary exposure likely contributes to the PAH biomarker levels in blood and urine [44, 66–69] and these pathways likely contribute to the internal PAH dose in the coke oven workers. PAH is likely both present in the particulate phase and in the non-particulate gas phase and both PAH fractions contribute to the amount of inhaled PAH [70]. In a recent occupational exposure study among Polish coke oven workers, strong correlations between PAH levels in air (both pre- and post-shift) and urinary excretion of 1-hydroxypyrene [35]. Furthermore, the content of PAH in the particle phase correlates to total PAHs (gas and particle phase) when measured for a range of emission sources [36]; and, in chimney sweeps, PAH metabolites correlated positively with the percentage of soot sweeping suggesting that PAH markers were correlated to soot particles [37]. In addition, in animals, the acute phase response has been reported to be proportional with pulmonary neutrophil influx and with total pulmonary deposited particle surface area [23, 71, 72]. Thus collectively these data suggest that the association between PAH and SAA reflect an association between particulate air pollution and SAA.

There is some evidence in the literature for the induction of the acute phase response of both PAH and of particles. In humans PAH exposure has been shown to be associated with serum CRP [21]. Biomonitoring studies having reported a correlation between occupational exposure to different types of particles and blood levels of acute phase proteins include the following exposures: Welding [73, 74], paper mill dust [75], emissions from firing of small weapons [76], and organic dust [53]. Increased blood levels of CRP has in some investigations been demonstrated in humans exposed to particulate matter [77–81]. In addition, animal studies have demonstrated that particles administered by intratracheal instillation and inhalation increase acute phase gene expression both at the mRNA and protein levels [24–32]. Thus these data are in line with the current findings suggesting that SAA is associated to PAH exposure and possibly associated to PAH in particles. Prolonged acute phase response induction has been linked to cardiovascular disease [10, 82–84], and there is evidence that both PAH and particulate air pollution have been associated with increased risk of cardiovascular disease in epidemiological studies [7, 8]. In a recent study of chimney sweepers, associations between different urinary PAH metabolites and diastolic blood pressure were reported. In addition, chimney sweepers had increased blood levels of homocysteine, cholesterol, and high-density lipoprotein (HDL) as compared to the control group. The authors suggested that PAH is a driving force for developing cardiovascular disease [37]. Coke oven emissions have been reported to be a risk factor for hypertension and abnormal electrocardiograms in coke oven workers [85] and excess mortality has been observed in coke oven plant workers. Causes of this excess were cancer and cardiovascular diseases [86]. In light of this our data also add to the notion that there is an association between PAH and particle air pollution and increased risk of developing cardiovascular diseases.

## Conclusions

Our data suggest that urinary 1-hydroxypyrene, as biomarker of short-term PAH exposure, and the serum level of CRP, predict the serum levels of SAA in coke oven workers; And thus that exposure of coke oven workers to PAH can lead to increased systemic acute response and potentially increased risk of cardiovascular disease.

## Additional files

Additional file 1: **Table S1.** Predictors of SAA levels with CRP excluded from the analysis. Multiple linear regression analysis of influence of PAH exposure evaluated by urinary excretion of 1-hydroxypyrene, anti-B[a]PDE-DNA adduct levels in blood cells, GSTM1, smoking status and diet habits on serum SAA levels in coke oven workers (*n* = 87). (DOCX 14 kb)

Additional file 2: **Table S2.** Predictors of CRP levels with SAA excluded from the analysis. Multiple linear regression analysis of influence of PAH exposure evaluated by urinary excretion of 1-hydroxypyrene, anti-B[a]PDE-DNA adduct levels in blood cells, GSTM1, smoking status and diet habits on serum CRP levels in coke oven workers (*n* = 87). (DOCX 14 kb)

## Data Availability

The datasets used and/or analysed during the current study are available from the corresponding author on reasonable request.

## Abbreviations

Anti-B[a]PDE)-DNA: Anti-benzo[a]pyrene diolepoxide
BEI: Biological exposure index
CRP: C-reactive protein
CTPV: Coal tar pitch volatiles
CYP450: Cytochrome P450
ELISA: Enzyme-linked Immunosorbent assay
GSTM1: Glutathione S-transferase Mu 1
HPLC: High performance liquid chromatography
LMF: Mononuclear lymphocyte plus monocyte fractions
LOD: Level of detection
PAH: Polycyclic aromatic hydrocarbons
PCR: Polymerase chain reaction
RR: Relative risk
SAA: Serum amyloid A
TLV: Threshold limit value
