# Supplementary material for: Association between a urinary biomarker for exposure to PAH and blood level of the acute phase protein serum amyloid A in coke oven workers
Source: Environ Health. 2019 Sep 2;18:81. doi: 10.1186/s12940-019-0523-1 (PMC6721239; doi:10.1186/s12940-019-0523-1)
Supplement: Supplementary file 1 — Table S1. Predictors of SAA levels with CRP excluded from the analysis. Multiple linear regression analysis of influence of PAH exposure evaluated by urinary excretion of 1-hydroxypyrene, anti-B[a]PDE-DNA adduct levels in blood cells, GSTM1, smoking status and diet habits on serum SAA levels in coke oven workers (n = 87). (DOCX 14 kb) [file 12940_2019_523_MOESM1_ESM.docx]

**Additional file 1 Table S1** Predictors of SAA levels with CRP excluded from the analysis. Multiple linear regression analysis of influence of PAH exposure evaluated by urinary excretion of 1-hydroxypyrene, anti-B[a]PDE-DNA adduct levels in blood cells , GSTM1, smoking status and diet habits on serum SAA levels in coke oven workers (n=87).

|  | 1-hydroxypyrene | anti-B[a]PDE-DNA adducts | GSTM1^a^ | Smoking^b^ | Diet^c^ |  |
| --- | --- | --- | --- | --- | --- | --- |
| β^d^ | 12.5 | -25.1 | -95.9 | -11.1 | 24.3 |  |
| T^d^ | 0.73 | -0.82 | -2.22 | -0.27 | 0.58 |  |
| *p*-value^d^ | 0.47 | 0.41 | **0.03** | 0.79 | 0.56 |  |

^a^ GSTM1 genotypes was treated as dichotomous variables: GSTM1 =1 or 0, Active or *0/*0.

^b^ Smoking = 1 or 0, current smokers or non-smokers.

^c^ Diet = 1 or 0, charcoaled meat consumption more or less than once per week, respectively.

^d^ The F test gave an F of 1.08 and a *p ­*value of 0.38. β is the slope. T is the test statistics β/standard deviation of β.
