# Supplementary material for: Association between a urinary biomarker for exposure to PAH and blood level of the acute phase protein serum amyloid A in coke oven workers
Source: Environ Health. 2019 Sep 2;18:81. doi: 10.1186/s12940-019-0523-1 (PMC6721239; doi:10.1186/s12940-019-0523-1)
Supplement: Supplementary file 2 — Table S2. Predictors of CRP levels with SAA excluded from the analysis. Multiple linear regression analysis of influence of PAH exposure evaluated by urinary excretion of 1-hydroxypyrene, anti-B[a]PDE-DNA adduct levels in blood cells, GSTM1, smoking status and diet habits on serum CRP levels in coke oven workers (n = 87). (DOCX 14 kb) [file 12940_2019_523_MOESM2_ESM.docx]

**Additional file 2: Table S2** Predictors of CRP levels with SAA excluded from the analysis. Multiple linear regression analysis of influence of PAH exposure evaluated by urinary excretion of 1-hydroxypyrene, anti-B[a]PDE-DNA adduct levels in blood cells , GSTM1, smoking status and diet habits on serum CRP levels in coke oven workers (n=87).

|  | 1-hydroxypyrene | anti-B[a]PDE-DNA adducts | GSTM1^a^ | Smoking^b^ | Diet^c^ |  |
| --- | --- | --- | --- | --- | --- | --- |
| β^d^ | -0.04 | -0.05 | -0.25 | 0.11 | -0.01 |  |
| T^d^ | -0.84 | -0.52 | -1.70 | 0.82 | -0.06 |  |
| *p*-value^d^ | 0.40 | 0.60 | 0.09 | 0.41 | 0.95 |  |

^a^ GSTM1 genotypes was treated as dichotomous variables: GSTM1 =1 or 0, Active or *0/*0.

^b^ Smoking = 1 or 0, current smokers or non-smokers.

^c^ Diet = 1 or 0, charcoaled meat consumption more or less than once per week, respectively.

^d^ The F test gave an F of 0.87 and a *p ­*value of 0.50. β is the slope. T is the test statistics β/standard deviation of β.
